# Supplementary material for: Prevalence and associated factors of depression among stroke family caregivers on follow up at Jimma medical center, southwest, Ethiopia: A cross-sectional study
Source: PLoS One. 2025 Jun 30;20(6):e0307068. doi: 10.1371/journal.pone.0307068 (PMC12208473; doi:10.1371/journal.pone.0307068)
Supplement: Attachment — Submitted filename: Response to Reviewers.docx [file pone.0307068.s003.docx]

**Rebuttal letter**

Dear Editor,

We are grateful to the reviewer for the time taken to review the manuscript, and the useful comments they provided to improve the manuscript. We have responded to the entire reviewer’ comments point-by-point, highlighting the changes in the manuscript; and they are summarized below: Once again, we thank the reviewers for the useful comments and suggestions.

| Comments of the reviewers | Reply by the author(s) | Changes done on page number and line number |
| --- | --- | --- |
| Editor #1 |  |  |
| 1.Please ensure that your manuscript meets PLOS ONE’s style requirements, including those for the file naming. | The comment accepted and corrected accordingly. |  |
| 2. we suggest you thoroughly copyedit your manuscript for language usage, spelling and grammar. | Thank you for the comment raised. We try to correct spelling error and grammar for language usage as highlighted in the manuscript by consulting different language experts. |  |
| 3. relate with funding  a. please clarify the source of funding (financial or material support) for your study.  b. state what role the funder took in the study. If the funder had no role in your study please state “the funders had no role in study design, data collection and analysis, decision to publish, or preparation of the manuscript”  c. if any authors received a salary from any of your funders, please state which authors and which funders.  d. If you did not receive any funding for this study, please state: “the authors received no specific funding for this work.” | Comment accepted and corrected accordingly. | Page # 14 |
| 4.funding should not appear in the acknowledgement section or other areas of your manuscript. | Comment accepted and corrected accordingly. We have removed funding related statements from the whole manuscript and included in the cover letter. | Page # 14 |
| 5. we noted that you have stated that you will provide repository information for your data at acceptance. Should your manuscript be accepted for publication, we will hold it until you provide the relevant accession numbers or DOIs necessary to access your data. | Thank you for providing this information and we have already summited repository data, so that you can accept it for publication as it was mentioned in cover letter. |  |
| 6. your ethics statement should only appear in the method section of your manuscript | Thank you for the comment and it is corrected accordingly. | Page # 8 |
| Reviewer # 1 |  |  |
| The author did not make it clear who are the caregivers, the family? The wives? The paid nurses Or others, and how did the author those caregivers…. all accompanying individuals to the hospitals are not the caregivers… | Thank you for the comment raised by the reviewer. The comment accepted and corrected accordingly. | Page # 5, line No # 1 |
| PHQ 9 is meant for screening purposes, not for diagnosis. Then how the diagnosis of depression was made? | Actually, PHQ 9 used for screening purpose and severity rater and the comment accepted and corrected accordingly. But when we’re reporting the caregiver’s depression, we’re not saying depression disorder, but based on assessment tool those scored 10 and above were considered as depressed which doesn’t mean that they have depression disorder. This is also supported by the literatures | Page # 6, line No # 3 |
| poor social support is considered a predictor of depression, but is it for stroke patients or caregivers? | There might be an association between patients’ poor social support and caregiver depression, but in our study it caregivers poor social support was a predictor of caregivers’ depression | Page # 13, paragraph #3, line No # 1 |
| Having no health insurance is again a predictor of depression. This is not clear. Paid caregivers or family members. | As our study population was stroke family caregivers, we’re stating that having no health insurance was a predictor of caregivers’ depression. | Page # 13, paragraph # 2, line No # 1 |
| The author did not narrate the details of caregivers are they professionals?... nurse... | The comment accepted and corrected accordingly. | Page # 5, line No # 1 |
