# Supplementary material for: Prevalence and associated factors of depression among stroke family caregivers on follow up at Jimma medical center, southwest, Ethiopia: A cross-sectional study
Source: PLoS One. 2025 Jun 30;20(6):e0307068. doi: 10.1371/journal.pone.0307068 (PMC12208473; doi:10.1371/journal.pone.0307068)
Supplement: Attachment — Submitted filename: Response_to_Reviewers_auresp_2.docx [file pone.0307068.s004.docx]

**Rebuttal letter**

Dear Editor,

We are grateful to the reviewers for the time taken to review the manuscript, and the useful comments they provided to improve the manuscript. We have responded to the entire reviewer’ comments point-by-point, highlighting the changes in the manuscript; and they are summarized below: Once again, we thank the reviewers for the useful comments and suggestions.

| Comments of the reviewers and editors | Reply by the author(s) | Changes done on page number and line number |
| --- | --- | --- |
| Editor #1 |  |  |
| 1 Your sampling methodology is unclear on whether multiple participants (family members) were selected from the same patient household/family. You need to clarify accurately and clearly.  I also recommend a language and grammar check. | Thank you for your constructive comments. The comment is accepted and corrected accordingly. Regarding the case in which multiple caregivers were appear together, only one caregiver was selected based on lottery mothed, as it can be found on the manuscript. | Page # 5, line 111,112. |
| Reviewer #1 | Thank you for your constructive comments. The comment was accepted and corrected accordingly. |  |
| Thank you for response. the authors almost responded for the concerns but I have some questions: 1. What is the practice of health insurance in your set up? | Here, in our setup the purpose of health insurance is that; those with health insurance will have free services like: medication, hospital admission bed, and other payment related services. As economic burden is one of the risk factors for stroke caregiver depression, those with health insurance were less vulnerable for depression. So that, health insurance was considered as one of independent variable in our study as it can be supported by different literature. |  |
| 2. Is there any care giver who get paid? | As it is mentioned on the response for question # 1, our study was on stroke family caregivers and there is no any payment for the caregiving service of their relatives. And our study participants were limited to stroke family caregivers. |  |
| Reviewer #2: | Thank you for your constructive comments. The comment was accepted and corrected accordingly. |  |
| 1.The study has a focus; however, it lacks clarity on multiple dimensions (differentiation between variables relevant to stroke caregiver’s vs those of stroke survivors, page 1, keywords),  inclusion/exclusion criteria, lines; 95-98, 115,116, sampling techniques, lines 94-104). | #Regarding the sample size calculation, we took “p” 24.3%, from the study conducted among stroke family caregivers in Nigeria. Because we perceived that, the socio-economic status of this country is almost similar with ours.  #The variables were separated as that of patients and caregivers.  # Regarding sampling techniques, the comment was accepted and corrected accordingly. |  |
| 2. The data collection tool is a combination of multiple scales that address both stroke survivors and stroke caregivers as well which creates another site of confusion, lines 111-113. | The comment was accepted and corrected accordingly.  The data collection has discussed for the patients and caregivers separately. |  |
| 3. Finally, an in-depth proof reading is required to ensure English language errors are fixed, lines 69, 70,71,72,86. | The comment was accepted and corrected accordingly. |  |
