# Supplementary material for: Prevalence and associated factors of depression among stroke family caregivers on follow up at Jimma medical center, southwest, Ethiopia: A cross-sectional study
Source: PLoS One. 2025 Jun 30;20(6):e0307068. doi: 10.1371/journal.pone.0307068 (PMC12208473; doi:10.1371/journal.pone.0307068)
Supplement: Attachment — Submitted filename: Response_to_Reviewers_auresp_4.docx [file pone.0307068.s006.docx]

**Rebuttal letter**

Dear Editor and reviewers!

We are grateful to the reviewers and academic editor for the time taken to review our manuscript, and the useful comments they provided to improve the manuscript. We have responded to the entire reviewer’ comments point-by-point, highlighting the changes in the manuscript; and they are summarized below: Once again, we thank the reviewers for the useful comments and suggestions.

| Comments of the reviewers and editors | Reply by the author(s) | Changes done on page number and line number |
| --- | --- | --- |
| Reviewer # 4 and academic editor |  |  |
| 1.  Avoid italicizing your text in the abstract section unless particularly requested. | Thank you for your constructive comments. The comment was accepted and corrected accordingly. |  |
| 2. Consider the proper and consistent use of analysis method in your study. I think the difference between multivariate and multivariable logistic regression analysis be noted and used accordingly. | That’s good and constructive comments. We have corrected accordingly and it’s highlighted | Page # 14, line 280. |
| 3. Line (30): you can make it, the association between depression and associated factors was estimated using odds ratio with its 95%CI and p-value. | It's modified accordingly. | Line # 29-30 |
| 4.Line (32): a total of 306 family caregivers participated in the study, making a response rate of 98.7%. | The total sample size of this study was 310 as it’s mentioned on method part of the documents. Of which only 306 was participated in the study which make response rate 98.7%. |  |
| 5. Line (39): it is good to put the meaning of your finding in the conclusion. What does more than 1/3rd prevalent mean in the study’s context. It is also good to highlight the implication of this finding to the study’s context. | That’s good comment and we have modified accordingly | Line # 38-39 |
| 6. Line 57: It is a long-standing and weakening condition that has a profound negative effect on their families. It looks grammatically incorrect, please make sure the sentence is coherent with preceding one. | Modified and highlighted | Line #55-56 |
| 8.the other comments | Other comments are modified accordingly | Line # 165,200, 218, |
| 9.The background section of the abstract must show the burden of the problem, the gap, and the intention of this study. | Thank you for your constructive comments dear reviewer.  # As the abstract section of the manuscript shows the summary, we only included the world report as the burden of the problem. And we merge the background and objective of the study as highlighted | Line #20-22 |
| 10. The sample size you put under the method and result sections are the same; if so, why is the response rate 98.7%?  Correct the upper limit of the confidence interval for the factor "spending more than 13 hours per day on care (AOR = 5.72, 95% CI: 2.32-1.12)." Introduction | The total sample size was 310 as mentioned in the method part. Out of which 306 were involved in the study which make the response rate 98.7%  Comment accepted and corrected accordingly | Line# 108  Line# 36 |
| 11.The introduction section lacks critical components, and intensive revisions are mandatory. The figures (prevalence of depression among caretakers of stroke patients) from previous studies must be addressed in a global-to-local context approach. | we accepted the comments and addressed it accordingly. Regarding the magnitude of the problem, we have mentioned the global prevalence as the summary of the report. Otherwise to the best our knowledge there is no local finding and, even in the African continent only 1 report we had found and mentioned in discussion part.  We also try to address risk factors we got from literature and scientific finding, which doesn’t mean that all risk factors are addressed. As its difficult to address all at once additional study might be indicated making this as source. Otherwise, other comments regarding grammatical errors and ideas out of our title has been omitted | Line# 72, 81 |
| #11.The source and study population are not appropriately defined. You write parents, offspring, siblings, spouse, and other relatives are the sources population, and if you get two during the visit, you select one of them by lottery method. Imagine if the stroke patient is married and his partner and his relative come together for the follow-up visit their relative has a 50% chance to be included in this study. I think this is unacceptable because his partner lives with him and she suffers a lot more than the relative. So, from the outset, the lottery method must be for equal relationships; otherwise, the findings are either over or underestimated. You tried to see it as a factor; however, the sampling approach is unacceptable. Sampled and study populations are different; hence, you must modify your study population. | #Source population is to whom we generalize our finding and the study population is from which we select our study participant, thus we define accordingly.  # The reason why we used lottery method was just to avoid over/underestimation of our finding. If we select the one who spent more time with the patient obviously it will overestimate the finding, so this was the only chance we had to use.  # as mentioned earlier JMC was one of the organization giving service for more than fifteen million diversified population living in south western part of the country. This will somewhat representative, even though not as that of national level-based study. |  |
| #12. For example, the information under the data collection and procedure from lines 131 to 149 does not align with the title. You have to modify it with other appropriate headings. Moreover, how did you organize your tool? Several missed variables may affect depression, so why do you extensively include them from the outset? Some variables were not appropriately measured, e.g. How did you assess alcohol use? In general, the method section needs further detailed clarification. | Comments accepted and addressed accordingly  # comments regarding tools we have mentioned the psychometric properties of the tools we have used including the validation. But what we have mentioned in this manuscript was those variables statistically significant at binary multivariable regression model. Example alcohol, other substances were measured using adopted ASSIST substance use scale. Since substance were not significant according to our study we didn’t mention it in the manuscript. Otherwise, other comments are modified accordingly. | Line # 110, 126-149 |
| #13. Paraphrase the sentences and appropriately link the tables and figures; do not write as see Table 1 below; instead, put like (Table 1). Where is figure one? Modify fragmented paragraphs under the discussion section. The majority of the references are outdated; please replace them with recent ones | Comment accepted and modified  Regarding the reference we are obligated to use out of dated reference since we didn’t found literature inline with our study objective/title | Line # 203, 221,224-228, 263, 264, 269, 270, 275-279 |
