# Supplementary material for: Prevalence and associated factors of depression among stroke family caregivers on follow up at Jimma medical center, southwest, Ethiopia: A cross-sectional study
Source: PLoS One. 2025 Jun 30;20(6):e0307068. doi: 10.1371/journal.pone.0307068 (PMC12208473; doi:10.1371/journal.pone.0307068)
Supplement: Attachment — Submitted filename: Response_to_Reviewers_auresp_5.docx [file pone.0307068.s008.docx]

**Rebuttal letter**

Dear Editor and reviewers!

We are grateful to the reviewers and academic editor for the time taken to review our manuscript, and the useful comments they provided to improve the manuscript. We have responded to the entire reviewer’ comments point-by-point, highlighting the changes in the manuscript; and they are summarized below: Once again, we thank the reviewers for the useful comments and suggestions.

| Comments of the reviewers and editors | Reply by the author(s) | Changes done on page number and line number |
| --- | --- | --- |
| Reviewer # 4 and academic editor |  |  |
| 1.  Please review your reference list to ensure that it is complete and correct. If you have cited papers that have been retracted, please include the rationale for doing so in the manuscript text or remove these references and replace them with relevant current references. | Thank you for your constructive and on time comments. The comment was accepted and corrected accordingly. | Reference # 2, 3, 5, 7, 8, 9, 11, 14, 15, 18, 23, 25, 28, 29, 30, 33, 37, 39, 41 |
| 2. comments of academic editor regarding grammatical and structural correction of sentences. | That’s good and constructive comments. We have corrected accordingly and it’s highlighted | Line # 20, 23, 24, 39, 80, 182, 195, 251, 256 and 275 |
| 3. Line #276: Some of the information was gathered from the patient's hospital case folder, which is secondary data. Yes, using secondary data can be a limitation, but it is not necessarily a limitation in and of itself. | Actually, what we have gathered from patients’ folder was diagnosis, comorbid medical illness, type of stroke and other clinical factors. We put our concern as limitation only because of secondary data. otherwise, we didn’t face any missing data, gaps etc. so we have removed that we have considered as limitation previously. |  |
