# Supplementary material for: Prevalence and associated factors of depression among stroke family caregivers on follow up at Jimma medical center, southwest, Ethiopia: A cross-sectional study
Source: PLoS One. 2025 Jun 30;20(6):e0307068. doi: 10.1371/journal.pone.0307068 (PMC12208473; doi:10.1371/journal.pone.0307068)
Supplement: Attachment — Submitted filename: Response_to_Reviewers_auresp_6.docx [file pone.0307068.s009.docx]

**Rebuttal letter**

Dear Editor and reviewers!

We are grateful to the reviewers and academic editor for the time taken to review our manuscript, and the useful comments they provided to improve the manuscript. We have responded to the entire reviewer’ comments point-by-point, highlighting the changes in the manuscript; and they are summarized below: Once again, we thank the reviewers for the useful comments and suggestions.

| Comments of the reviewers and editors | Reply by the author(s) | Changes done on page number and line number |
| --- | --- | --- |
| Comments raised by academic editor regarding typing, punctuation and grammatical error | Thank you for your constructive and on time comments. The comment was accepted and corrected accordingly. | Line# 24, 28, 31, 84, 88, 89 and 115 |
| Reviewer # 5 | We have great thanks for the comments and the comment was accepted and corrected accordingly. | Line # 69, 72, 75, 77, |
|  | Comment regarding line # 78 was taken to the beginning of introduction part. | Line # 68 |
|  | The background section has been modified as its highlighted. |  |
|  | Some typing and grammatical error has been corrected accordingly | Line # 87, 94, 97, and 115 |
| Reviewer # 6 | Thank you for the guiding comments you gave us. For the comments regarding to typing and grammatical error we tried to correct as highlighted. | Line# 24, 28, 31, 75, 84, 88, 89 and 115 |
|  | We have used binary logistic regression analysis and it is mentioned throughout the document. |  |
|  | We presented the bivariable and multivariable result with single table, because it is recommended previously to merge the tables. To identify the result of each binary logistic analysis, its indicated separately by single and double “*” at the bottom of the table. |  |
