# Supplementary material for: Prevalence and associated factors of depression among stroke family caregivers on follow up at Jimma medical center, southwest, Ethiopia: A cross-sectional study
Source: PLoS One. 2025 Jun 30;20(6):e0307068. doi: 10.1371/journal.pone.0307068 (PMC12208473; doi:10.1371/journal.pone.0307068)
Supplement: Attachment — Submitted filename: Response_to_Reviewers_auresp_7.docx [file pone.0307068.s010.docx]

**Rebuttal letter**

Dear Editor and reviewers!

We are grateful to the reviewers and academic editor for the time taken to review our manuscript, and the useful comments they provided to improve the manuscript. We have responded to the entire reviewer’ comments point-by-point, highlighting the changes in the manuscript; and they are summarized below: Once again, we thank the reviewers for the useful comments and suggestions.

| Comments of the reviewers and editors | Reply by the author(s) | Changes done on page number and line number |
| --- | --- | --- |
| Comments raised by academic editor on spelling and grammatical | Thank you for your constructive and on time comments. The comment was accepted and corrected accordingly. | Line# 25, 28, 85-90 |
|  | Regarding sample size calculation we didn’t calculate sample size for associated factors considering that it’s optional. This can be taken as a limitation |  |
| Reviewer # 7 | Dear reviewer we have great thanks for the comments you have raised and we try to address one by one. Regarding to spelling error the comment was accepted and corrected accordingly. Regarding the prevalence the previous reviewers commented me to include the prevalence in abstract part. | Line # 25, 26 and 38-40 |
|  | Comments in introduction part regarding abusive words and grammatical error was corrected accordingly | Line # 81, 85-89 |
|  | Methodology  #Regarding this study, up to this research was conducted no published research conducted in our country regarding this title, thus being the first finding for this country may fill the gaps in our country and that is why we used the Nigerian finding. #Even most of the researches done outside the country were conducted before five years.  #The reason why we include the caregivers who at least spent two weeks was; in order to diagnose depression at least two weeks is mandatory, even if this is for screening purpose.  # Kinship relation was one of the factors that we have used as independent factor and associated with depression at bivariable binary logistic regression, but fail to associate at final model. |  |
| Reviewer # 7 | Discussion  We accepted the comments and try to address and modify the discussion parts |  |
