# Supplementary material for: Prevalence and associated factors of depression among stroke family caregivers on follow up at Jimma medical center, southwest, Ethiopia: A cross-sectional study
Source: PLoS One. 2025 Jun 30;20(6):e0307068. doi: 10.1371/journal.pone.0307068 (PMC12208473; doi:10.1371/journal.pone.0307068)
Supplement: Attachment — Submitted filename: Reviewer Comments to authors_R5.docx [file pone.0307068.s007.docx]

**Reviewer comments to authors**:

The authors structured the paper nicely to assess the prevalence and associated factors of depression among stroke family caregivers on follow-up at Jimma Medical Center, southwest Ethiopia. I found that this paper intends to fill a gap in the literature related to depression among stroke family caregivers on follow-up. However, I found some issues discussed below:

1. **In the introduction:**
   - There are grammar and/or typing errors in your manuscript. For instance, on line 75, "hahigher..." Please correct it with “have a higher.”.
2. **In the methods:**
   - There are many kinds of logistic regression models. I realized that you have used binary logistic regression. Thus, please replace logistic regression by binary logistic regression analysis in your manuscript.
3. **In the results**:
   - On Table 2, have you presented the results of bivariable and multivariable binary logistic regression analyses together? What does it mean? Please correct the table. If it’s the result of multivariable binary logistic regression analysis, say, “Table 2: Multivariable binary logistic regression analysis for factors associated with depression among family caregivers of patients with stroke follow-up treatment at Jimma Medical Center, southwest Ethiopia, 2021.”.
   - What does independent factor mean? Please say either independent variables or factors.
